# Clear Cell Sarcoma of Kidney in a Neonate

**Published:** 2014-07-10

**Authors:** Avinash SukdevJadhav, Sanjay Jain, Neeraj Tuteja, Leeladhar Agrawal

**Affiliations:** Sir Padamat Mother and Child Health Institute, Sawai Man Singh Medical College and Hospital, Jaipur

**Keywords:** Clear cell sarcoma, Kidney, Neonate

## Abstract

A neonate presented with abdominal mass in left flank was investigated and operated upon. Histopathology confirmed the diagnosis of clear cell sarcoma of the kidney. Post-operatively, chemotherapy was given according to the NWTS-5 protocol. During follow-up, the patient has shown good recovery after 7 months of surgery.

## INTRODUCTION

Wilm's tumors comprise 6% to 7% of childhood cancer; whereas the remaining non-Wilm's renal tumors comprise less than l% [1]. In neonates amongst non-Wilm’s tumors, most common are congenital mesoblastic nephroma (CMN), rhabdoid tumor of the kidney, and multilocular cystic nephroma, nephroblastomatosis complex and ossifying renal tumor of infancy. CCSK is most commonly observed in the age group of 3 to 5 years [2]. It is the most frequently misdiagnosed renal tumor in children, as it is unusual, has varied morphology, and there are no specific diagnostic markers [3]. Here we report a case of CCSK in a neonate.

## CASE REPORT

A 28-day-old male neonate was hospitalized with progressive increasing abdominal mass for last 2 weeks. On palpation, a hard lump was noted in left flank. Ultrasound abdominal showed the mass of renal origin; there was no vascular involvement or lymphadenopathy. Routine investigations were within normal limits. Computed tomographic (CT) scan revealed left renal mass of heterogeneous nature along with cystic areas. X-ray of the chest was normal. Left nephrectomy was done and the specimen was examined histopathologically. Cord cells and spindle shaped cells interspersed in fibrovascular matrix arranged in storiform pattern were seen. Diagnosis of clear cell sarcoma of kidney was made. Skeletal survey was done after confirmation of histology to rule out bone metastasis. Depending on histopathology report and work up for metastasis patient was found to have Stage 1 disease. Chemotherapy according to regimen I of NWTS-5 protocol was administered for 24 weeks. Patient was examined on his each hospitalization for chemotherapy schedule. There were no significant complications due to chemotherapy. Radiotherapy was not given. Contrast enhanced CT of the abdomen and pelvis was repeated. X-ray chest and skeletal survey were done after 3 and six months to rule out recurrence. There were no signs of any residual disease on follow-up. 

**Figure:**
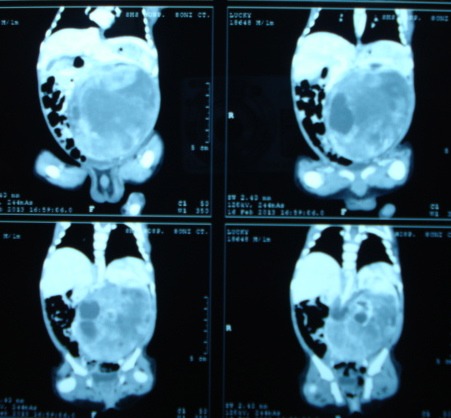
Figure 1: Left renal mass, heterogeneous in nature showing cystic areas.

**Figure:**
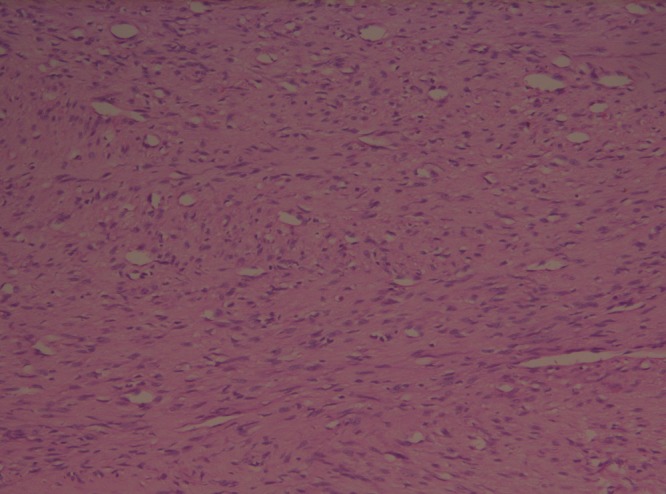
Figure 2: Histopathological picture revealing clear cells, spindle cells and fibrovascular septa.

## DISCUSSION

CCSK was first described in 1978 by three distinct groups, Beckwith and Palmer, Morgan and Kidd, and Marsden and co-workers who named it “bone metastasizing renal tumor of childhood” since it has predilection for skeletal metastasis [1]. Bone metastases occur in 40% to 60% of patients with CCSK, unlike less than 2% of patients with Wilms’ tumor. Other clinical features, which separate it from Wilm’s tumor, are a lack of association with sporadic aniridia and hemihypertrophy [1]. The peak incidence is between 3 and 5 years of age. It is very rare in infants younger than 6 months [4]. The present case is of a neonate. Ultrasonography is the initial investigation, which showed the mass was heterogeneous in echogenicity with cystic components and necrosis. On CT scan, non-homogeneous enhancement, with attenuation less than that of normal renal parenchyma is usually seen. Also, these tumors may contain areas of low attenuation corresponding to necrosis and cysts [5]. On cut section, these tumors appear grossly as tan-gray, soft, and mucoid. Cystic foci are almost always present and may represent the dominant feature, resembling that of multilocular renal cyst. On histopathological examination, CCSK has three components, namely, (1) cord cells, (2) septal cells, (3) an intercellular matrix of mucopolysaccharide. Cord cells are small round to oval shaped cells with mitotic figures. Septal cells are spindle shaped cells seen surrounding the fibrovascular septa. The matrix of mucopolysaccharide broadcasts the clear appearance of CCSK. 

There are four modalities of treatment surgical resection, surgical resection with chemotherapy, surgical resection with radiotherapy, surgical resection with chemotherapy and radiotherapy. For unexplained reasons, CCSK has been known to have the best outcome amongst all perinatal tumors [6].

Radical nephrectomy is the initial treatment of choice if the lesion is resectable [5]. It has been found that in the NWTS-3, the addition of doxorubicin to the combination of vincristine, dactinomycin, and radiation therapy resulted in an improvement in disease-free survival in patients with clear cell sarcoma of the kidney [7]. Siebel et al found that in NWTS-4, patients treated with vincristine, doxorubicin, and dactinomycin for 15 months had an improved relapse-free survival rate compared with patients treated for 6 months (87.5% vs 60.6% at 8 y). Also, the overall survival has improved for patients with clear cell sarcoma of the kidney from NWTS-3 to NWTS-4 (83% vs 66.9% at 8 y) [8]. In the NWTS-5 protocol, CCSK is treated with radical nephrectomy followed by chemotherapy with vincristine, Doxorubicin and Etoposide for 24 weeks and radio- therapy. Overall survival is 69% [9]. Four important prognostic factors are treatment with Doxorubicin, stage of the disease, age at the time of diagnosis and presence of tumor necrosis [7].

Emphasis should be given to look for necrosis in the radiological investigations in such cases as presence of necrosis is one of the prognostic indicators. Survival in this case can be attributed to earlier detection and lower stage of the disease at time of diagnosis.

## Footnotes

**Source of Support:** Nil

**Conflict of Interest:** None

**Editorial Comment:** In the largest SIOP cohort described so far, the median age at diagnosis for CCSK was 2.6 years [1]. Stage IV disease and young age were significant adverse prognostic factors for event-free survival (EFS), whereas factors such as gender, tumour volume and type of initial treatment were not found to be prognostic for EFS and overall survival (OS) [1]. Survival of a neonate with CCSK therefore is worth lauding. As such in the world literature, less than a dozen cases of CCSK have been reported in the fetuses and the neonates. In 2008, the celebrated pediatric pathologist Issac H Jr. in a review of 210 renal reported tumors diagnosed between 1960 and 2007 in 47 fetuses and 163 infants less than 2 months olds had reported only 7 cases of CCSK [2]. On review of the literature in 2005, Hung had found only 7 cases of CCSK aged less than 6 months of age (including one fetal case that he reported) of which two were extrarenal cases [3]. Although the authors have quoted that for unexplained reasons, CCSK has been known to have the best outcome amongst all perinatal tumors [2], it is hard to fathom. One issue that needs to be understood is that the neonates would tolerate chemotherapy poorly; the authors were lucky in the index case. It would be prudent to restrict adjunct therapy to single agent chemotherapy, e.g., vincristine [4], and to avoid radiotherapy for such young presenters of CCSK.

References

1. Furtwängler R, Gooskens SL, van Tinteren H, de Kraker J, Schleiermacher G, Bergeron C, et al. Clear Cell Sarcomas of the Kidney registered on International Society of Pediatric Oncology (SIOP) 93-01 and SIOP 2001 protocols: A report of the SIOP Renal Tumour Study Group. Eur Cancer, 2013: 49: 3497-3506.

2. Isaacs H Jr. Fetal and neonatal renal tumors. J Pediatr Surg. 2008; 43:1587-95.

3. Hung NA. Congenital "clear cell sarcoma of the kidney". Virchows Arch. 2005;446:566-8.

4. Suzuki H, Honzumi M, Itoh Y, Umehara N, Moriyama S, Funada M. Clear-cell sarcoma of the kidney seen in a 3-day-old newborn. Z Kinderchir. 1983; 38:422-4.

